# Systems Responses to Progressive Water Stress in Durum Wheat

**DOI:** 10.1371/journal.pone.0108431

**Published:** 2014-09-29

**Authors:** Dimah Z. Habash, Marcela Baudo, Matthew Hindle, Stephen J. Powers, Michael Defoin-Platel, Rowan Mitchell, Mansoor Saqi, Chris Rawlings, Kawther Latiri, Jose L. Araus, Ahmad Abdulkader, Roberto Tuberosa, David W. Lawlor, Miloudi M. Nachit

**Affiliations:** 1 Plant Biology and Crop Science, Rothamsted Research, Harpenden, United Kingdom; 2 Computational and Systems Biology, Rothamsted Research, Harpenden, United Kingdom; 3 Laboratoire D'agronomie, National Agricultural Research Institute of Tunisia, Ariana, Tunisia; 4 Dept. of Vegetal Biology, Faculty of Biology, Barcelona, Spain; 5 Biotechnology Department, General Commission for Scientific Agricultural Research, Damascus, Syria; 6 Dept. of Agroenvironmental Science and Technology, University of Bologna, Bologna, Italy; 7 Biodiversity and Integrated Gene Management Program, International Center for Agricultural Research in the Dry Areas, Rabat, Morocco; Institute for Sustainable Agriculture (IAS-CSIC), Spain

## Abstract

Durum wheat is susceptible to terminal drought which can greatly decrease grain yield. Breeding to improve crop yield is hampered by inadequate knowledge of how the physiological and metabolic changes caused by drought are related to gene expression. To gain better insight into mechanisms defining resistance to water stress we studied the physiological and transcriptome responses of three durum breeding lines varying for yield stability under drought. Parents of a mapping population (Lahn x Cham1) and a recombinant inbred line (RIL2219) showed lowered flag leaf relative water content, water potential and photosynthesis when subjected to controlled water stress time transient experiments over a six-day period. RIL2219 lost less water and showed constitutively higher stomatal conductance, photosynthesis, transpiration, abscisic acid content and enhanced osmotic adjustment at equivalent leaf water compared to parents, thus defining a physiological strategy for high yield stability under water stress. Parallel analysis of the flag leaf transcriptome under stress uncovered global trends of early changes in regulatory pathways, reconfiguration of primary and secondary metabolism and lowered expression of transcripts in photosynthesis in all three lines. Differences in the number of genes, magnitude and profile of their expression response were also established amongst the lines with a high number belonging to regulatory pathways. In addition, we documented a large number of genes showing constitutive differences in leaf transcript expression between the genotypes at control non-stress conditions. Principal Coordinates Analysis uncovered a high level of structure in the transcriptome response to water stress in each wheat line suggesting genome-wide co-ordination of transcription. Utilising a systems-based approach of analysing the integrated wheat’s response to water stress, in terms of biological robustness theory, the findings suggest that each durum line transcriptome responded to water stress in a genome-specific manner which contributes to an overall different strategy of resistance to water stress.

## Introduction

Durum wheat is a major staple crop in the Mediterranean basin as a source of semolina for the production of pasta, couscous, burghul and other local products. It is cultivated primarily under rain-fed conditions across a wide range of environments varying in the profile and quantity of rainfall with drought as a major abiotic stress [1], [2]. Regional to global-scale climate models predict, with high confidence, that the Mediterranean region will face soil drying in the future as temperatures rise and this may increase incidents of agricultural drought [3], [4], [5]. This will have serious negative implications for crop productivity and food security and thus crop yield and its component traits are targets for wheat breeders in their efforts to design cultivars for drought-prone environments as a means to ameliorate the climatic problems [6]. However, crop yield and drought resistance are complex genetic traits exacerbated by the fact that drought is often encountered with other abiotic and biotic constraints and these factors have contributed to the slow progress, so far, in breeding for relative drought resistance.

To address these challenges, various national and international efforts have been proposed to enhance wheat production by integrating technologies and approaches in plant science to underpin breeding efforts [7]. Thus, improving our understanding of the crop’s responses to drought stress has emerged as a major target. Genetic studies on durum wheat have identified quantitative trait loci (QTL) for yield and yield component traits as a first important step in understanding and deconstructing the complex genetic factors defining drought resistance [8], [9], [6]. In parallel, studies of plants exposed to water stress under controlled environmental conditions enable the mechanistic understanding of the complex responses, adaptations, acclimation and recovery from stresses at the molecular, biochemical and physiological levels [10], [11], [12]. Genomic tools and ‘omic’ technologies have recently been employed to screen for the genes, proteins and metabolites responsive to water stress, and have identified key molecular elements [13], [14]. Physical stress signals activate a series of molecular cascades and signalling components, both abscisic acid (ABA) and non-ABA-dependent, responsive to abiotic stresses in plants [15]. Common features have been identified in Arabidopsis and grass species [16] which have enabled the initial modelling of gene network responses under water stress [17]. These studies form a mechanistic basis for our knowledge of how physical signals are transduced to biochemical processes which ultimately translate into adjustments of metabolism and physiology to stress. Whilst genetic manipulation of key candidate genes, arising from such studies, has resulted in some form of resistance to water stress in individual transgenic plants, transferring such benefits from model species to improve crop performance under drought has been markedly less successful [18]. There is an urgent need to improve our understanding of drought effects on crops by combining comparisons of cultivars of different field performance with detailed analysis of their resistance mechanisms under well-defined plant water deficits. In addition, it is increasingly argued that systems-based approaches are necessary to integrate knowledge across genetics, genomics, breeding and plant physiology to tackle the complex genetic traits defining drought resistance [19], [20], [21]. Focusing system studies on crop species *per se* is also advocated considering the importance of genetic backgrounds and species-specific differences in adaptations to stress and for the immediate development of drought resistant crop cultivars, as demonstrated in bread *versus* durum wheat by [22].

Durum wheat grown in the Mediterranean is susceptible to post-anthesis terminal drought, a phase of growth which directly affects yield. Thus wheat breeders have targeted this type of drought by selecting for cultivars with a fast growth rate that rapidly accumulate biomass before flowering as a water stress avoidance strategy [2]. In addition, they have also developed wheat germplasm of promising yield potential by applying a double gradient selection strategy under both scarce and optimal water environments [8]. These efforts have been relatively successful in terms of yield improvement but the physiological and molecular basis of these drought resistant cultivars remains complex and unclear. Our aim in this study was to dissect the responses of drought resistant durum breeding lines to water stress in an attempt to identify molecular and physiological properties defining stress resistance and thus to build knowledge to accelerate the breeding effort. We chose wheat lines from the breeding programme at the International Centre for Agricultural Research in the Dry Areas (ICARDA) with good yield under irrigated and drought prone environments established in multi-year trials in the field (unpublished observations). Our study targets the post-anthesis developmental phase which corresponds to the period when plants are susceptible to terminal drought under field conditions in the Mediterranean basin. We applied rigorous criteria for the development of the water stress experiments, both in design and measurements, following [23]. High-throughput gene expression technology was applied in parallel with physiological measurements to profile durum lines at a range of water deficits (water stress transients) designed to dissect the systems responses to stress. Mathematical and bioinformatics methods were used to deconstruct the complex datasets and results are presented in formats amenable for further mining and exploration. Analysis was undertaken on datasets at equivalent leaf relative water content (RWC) to establish evidence of differential plant genome stress responses amongst the three wheat lines. Results demonstrate a global transcriptome response to water stress in all three lines that has a systems feature of high levels of coordination and uncovered within that key genome-specific differences. These results are discussed using a systems-based framework of biological robustness theory and how it could impact the search for new breeding strategies for drought resistance in durum wheat.

## Materials and Methods

### Plant material

Two durum wheat [*Triticum turgidum* L. ssp. *durum* (Desf.) Husn.] parents plus one recombinant inbred line (RIL) from the Cham1×Lahn mapping population developed at ICARDA were selected for this study because of their combined drought resistance and yield potential established in multiple year × site field trials in the Mediterranean region. Lahn was characterised by high yield potential under favourable rain environments, Cham1 exhibited relatively higher yield under drought, and RIL2219 was established as being more drought resistant than the best parent in addition to showing good yield stability (unpublished observations). Data collected in multiple field trials (30 over four years) showed RIL2219 closer to Cham1 in its overall phenology so that it reached heading 1.2 and 6 days earlier, on average, than Cham1 and Lahn respectively; it’s grain fill duration was 1.69 and 2.69 days later than Cham1 and Lahn respectively; and it was similar to cham1 for days to maturity with both reaching it 5 days earlier than Lahn (unpublished observations).

### Plant growth and experimental design

Seeds were sown in peat-free soil-based compost enriched with slow release fertilizer (Osmocote, Scotts UK Professional, Ipswich, Suffolk) and seedlings were vernalized for 3 weeks to synchronise growth and flowering. Three plants per line were transplanted into 3.5 L pots and grown under controlled chamber conditions of 25°C day/20°C night, 14 hours photoperiod, 800 µmol^−2.^s^−1^ photon flux density and 65% relative humidity. Pots were watered uniformly each day until the start of the experiment. Plants were arranged using a randomised block design with 3 biological replicates per combination of line, treatment (control or stressed) and time point of the stress. At anthesis, plants were subjected to four independent time series water deficit experiments (termed water stress transients) by withholding water from pots for a period of up to 6 days with parallel controls being well watered, all measurements were taken at 10 am on a daily basis following the initiation of stress. Flag leaves were sampled from 3 experiments for molecular and biochemical studies and from one representative experiment for photosynthetic studies, one week after flowering (Zadoks scale 70). For each experiment, a flag leaf was sampled for RNA extraction and ABA content, another was taken for photosynthetic and osmotic adjustment measurements and a third for leaf relative water content (%RWC) and water potential.

### Measurements of plant water status

The water status of the plants was monitored by flag leaf % RWC (fresh mass – dry mass)/(turgid mass – dry mass) × 100), following [24] and leaf water potential was measured with a pressure chamber, after [25]. Difference between pot mass in the morning and afternoon gave the total plant plus soil water loss.

### Physiological and biochemical measurements

Flag leaf transpiration rate, stomatal conductance to water, and CO_2_ assimilation rate were measured at 800 µmol m^2^ s^−1^ PFD using portable infrared gas analysers (LICOR LI-64 LI-COR Biosciences, Nebraska, USA and SIRAS PP Systems, Norfolk, UK). Plant height, tiller number, development stage and leaf area were measured to monitor growth. Osmotic potential and adjustment was measured using a vapour pressure Osmometer (5100C, Wescor, USA) on samples of sap from frozen, thawed, macerated and centrifuged leaves. ABA was quantified by GC-MS, essentially as described in [26], except that freeze-dried leaf samples were homogenised in 80% methanol-water using a Polytron homogeniser, after which [3-methyl-^2^H_3_]ABA (20–25 ng) was added as internal standard.

### RNA isolation and array hybridisation

Individual flag leaves, one leaf per biological sample, were sampled for RNA extraction and transcript expression studies. For all three wheat lines, 3 biological replicates per line were taken at days 1, 2, 3, 4, 5 (all 3 lines) and 6 (RIL2219 only) of the stress plus day 0 and 1 from the well watered controls to give a total of 66 samples. Leaves were frozen in liquid N_2_ and total RNA was extracted using TRIZOL Reagent (Invitrogen, UK) following the manufacturer’s recommendations. Total RNA was DNase treated with TURBO DNase enzyme (Ambion) and cleaned using RNeasy columns (Qiagen) following the manufacturer’s instructions. High quality RNA was submitted to transcriptome profiling at Bristol University (http://www.bristol.ac.uk/biology/research/transcriptomics/). Expression analysis was carried out on Affymetrix GeneChip Wheat genome arrays using Affymetrix GeneChip Eukaryotic One cycle target labelling reagents and protocols (701028 Rev 4). Five µg of total RNA was first reverse transcribed using a T7-Oligo(dT) promotor primer in a first strand cDNA synthesis reaction, followed by RNase H mediated 2nd strand cDNA synthesis. The double stranded cDNA was purified and served as a template in a subsequent in vitro transcription (IVT) reaction carried out in the presence of T7 RNA polymerase and a biotinylated nucleotide analog/ribonucleotide mix to produce complimentary biotinylated RNA (cRNA). The biotinylated cRNA targets were cleaned up, fragmented and hybridised to Wheat Genome arrays for 16 hours in a GeneChip Affymetrix Hybridisation oven 640. Hybridised probe arrays were then washed and stained with a streptavidin-conjugated fluorescent staining solution followed by antibody amplification of the fluorescent signal in a GeneChip fluidics 450 station using the appropriate fluidics scripts for the array format. Following washing and staining, arrays were scanned using the GeneChip scanner 3000. Cell intensity data was automatically computed from the image data using GeneChip Operating Software (GCOS) under default settings. The quality and integrity of both total RNA and labelled and fragmented cRNA was assessed on an Agilent bioanalyser RNA Nano assay.

### Data processing and modelling

Microarray data are available in the ArrayExpress database (www.ebi.ac.uk/arrayexpress) under accession number E-MTAB-2410. Microarray data initial analysis was carried out using GenespringGX 8.0 (Agilent Technologies, Inc., Santa Clara, California, USA). The set of array raw data files (.cel files) were pre-processed using the gcRMA algorithm [27] to standardise the mean and variance of expression. Probesets were filtered to remove those with mean signal values less than 10, which were deemed not expressed. All probeset signal data (hereafter referred to as transcript or probe expression) from the filtered set were then exported for ANOVA and multivariate statistical analysis using GenStat (2010, thirteenth edition, VSN International Ltd, Hemel Hempstead, UK). To account for the time differences in wheat line responses to the stress and to present the transcript expression results in terms of equivalent leaf RWC, data were interpolated according to suggestions for handling results of time series experiments [28]. Firstly, the logistic relationship of RWC over time (days) was used to facilitate interpolation of transcript expression on the log_2_ scale with respect to leaf RWC at proposed levels of 90, 82, 74, 66, 58 and 50; these levels of RWC were chosen to reflect a range of water deficit, whilst maintaining 6 observed occasions. RWC was then modelled on time (days of stress), fitting a logistic curve to the RWC data collected from each wheat line, using nonlinear regression, RWC(*time*) = *a*+*b*/[1+ exp((*time* - *c*)/*d*)], for estimable parameters *a*, *b*, *c*, and *d*. For each transcript by replicate (statistical block) by wheat line combination, the change in transcript expression data over the time course was interpolated using Lagrange polynomials [29], [30] which enabled the transcript expression at the time points corresponding to the proposed RWC levels to be calculated. The results were further analysed by ANOVA and log_2_ transformation which considered the main effect of, and interaction between, the line and RWC factors, and also accounted for the block effect. The transcripts were filtered into groups given significant F-test (p<0.01, to ensure well-defined groups) results, after correcting p-values for multiple testing and false-discovery [31] and least significant difference (LSD) values (p = 0.05) were used to compare means. Principal Coordinates Analysis (PCo) was applied to the RWC-interpolated data for the 19062 probes derived from 54 samples (three replicates of each of six RWC levels- 0–5 days- by three wheat lines) [32]. The probes proposed to be responsible for the separation observed in a given dimension were found by correlating the coordinates of the points in that dimension to transcript expression values, using an approximate F-test and extracting those transcripts with the greatest (most statistically significant) F-values. Physiological and biochemical measures were modelled with respect to RWC using linear or non-linear regression, testing whether single, parallel or separate lines (or curves) were required (F-tests) for the wheat lines. Linear regression was used for leaf water potential, water loss and osmotic adjustment. Gompertz curves, of the form *y* = *C* – *C*[exp(exp(−*k*(*RWC* − *m*))], were used for rate of CO_2_ assimilation, transpiration rate and stomatal conductance, with parameters *C* being the asymptote, *m* the inflection point and *k* the exponential rate separate, or common, for each wheat line. ABA content showed an asymmetric rise to a peak, therefore a rational functions model was used, log(ABA) = *a*+[(*b*+*cRWC*)/(1+ *dRWC*+*fRWC* ^2^)], with estimable parameters *a*, *b*, *c*, *d* and *f*, taking ABA on the log (to base *e*) scale to account for greater variability in this measure.

To estimate the contribution of allelic differences in gene sequences to probe hybridisation, for each perfect match probe the significance of the ratio changes across lines was calculated in proportion to the corresponding mean probe-set abundance. Robust Multiarray Averaging (RMA) background correction and constant normalisation was applied to the probe intensities [33], [27]. The probe intensities were transformed using the log_2_ of the ratio of the probe to the mean of the corresponding probe-set intensity. For probe-set intensities the same probe normalisation and background corrections were applied, together with perfect match correction and summarization using median polish [33], [27]. The significance of the presence of allelic difference for lines was determined from two-way ANOVA of line and time factors, using the p-value for the significance of the difference across lines.

### Annotation and MapMan pathways analysis

The wheat Affymetrix chip was designed with 11 probe sets per gene and the majority of the analysis in this study focused on the average expression value of the 11 probes in a probe set. In our analysis, we did not filter out any probes or identify unigenes, thus we present results for each probe set signal intensity that represent changes that could be due to unigenes, splice variants, homeoalleles or several members of a gene family. Probes were assigned to orthologous genes and metabolic bins, or pathways, using the MapMan programme following the standard protocol [34], [35], bin # 35 was unassigned annotation. When referring to probe intensity or transcript expression of genes from a particular metabolic pathway, from now onwards in the text, this refers to an annotated orthologous gene. qPCR was done on eight genes and results showed similar changes to Affymetrix probe expression as a function of the stress transient in all lines (unpublished observations).

## Results

### Physiological responses of wheat lines to water stress

**Figure 1:**
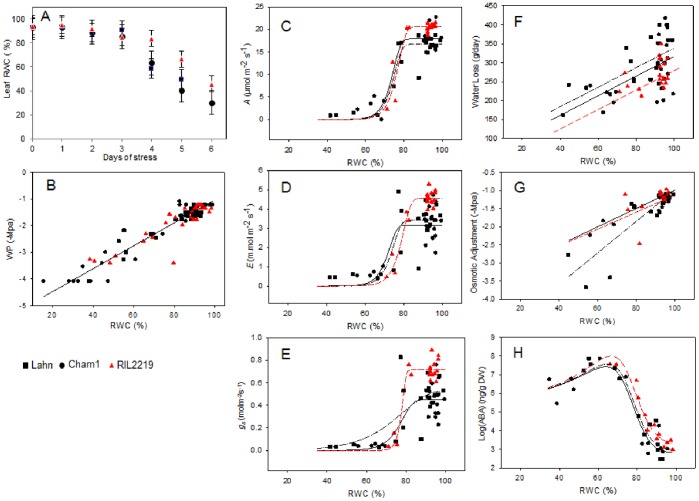
Physiological and biochemical parameters of leaves during water deficit. Plant water status was measured by flag leaf %RWC as a function of days of stress (A), leaf water potential (B) and total plant water loss (F) during a stress transient of 0–6 days for three wheat lines Lahn (▪), Cham1 (•) and RIL2219 (▴). Leaf photosynthetic and biochemical parameters were CO_2_ assimilation (C), transpiration (D), stomatal conductance (E), osmotic adjustment (G) and ABA content (H). Linear regression analysis gave a single line relationship for leaf water potential, parallel lines (p<0.05, F-test) for water loss, and separate lines (p<0.05, F-test) for osmotic adjustment. Using the Gompertz curve, separate *C* and *k* parameters (p<0.05, F-tests) were required for CO_2_ assimilation, whereas for transpiration and stomatal conductance only separate *C* parameters (p<0.05, F-test) were required; (C–H) red line, black dashed line and black solid line are for RIL2219, Lahn and Cham1 respectively. For ABA, separate c parameters were significant (p<0.05, F-test) in the rational functions model.

Cessation of watering and loss of water from the soil for 6 days progressively decreased flag leaf RWC and water potential for all wheat lines (Figure 1 A): the initial RWC was identical for the three cultivars. It was not possible to accurately measure RWC for Lahn on day 6 as it was severely wilted. RWC decreased more slowly in RIL2219 than in either parent, as demonstrated by its larger RWC after day 3, related to the slower rate of total water loss from its pots both as a function of time and of RWC (Figure 1A, 1F). The relation between RWC and water potential of the flag leaves was linear, and identical, in the three cultivars (Figure 1B) and therefore RWC was used as the reference for tissue water status. As the rates of water loss and decrease in RWC differed significantly between the cultivars, all subsequent analyses were made with reference to RWC. RIL2219 had higher photosynthetic rate (Figure 1 C), instantaneous transpiration rate (Figure1 D) and a larger stomatal conductance (Figure 1 E) in the early phases of water depletion (from ca. 100 to 80% RWC). However, these decreased substantially in all cultivars (and marginally more so in RIL2219) as RWC fell from ca. 80 to 60% and where we also observed that RIL2219 and Cham1 maintained a higher osmotic adjustment for equivalent RWC when compared to Lahn (Figure 1 G). Furthermore, the ABA content of turgid leaves was larger in RIL2219 than Cham1 or Lahn notably at the start of the experiments in leaves at equivalent RWC, but increased similarly as RWC decreased, rising greatly and reaching a maximum at about 65% (Figure 1 H) after which there was a general decline. There were no statistically significant (p<0.05, F-test) difference in flowering time, leaf area or plant height between the lines in any of the experiments (unpublished observations).

### Dissection of transcriptome responses to progressive water stress

**Figure 2:**
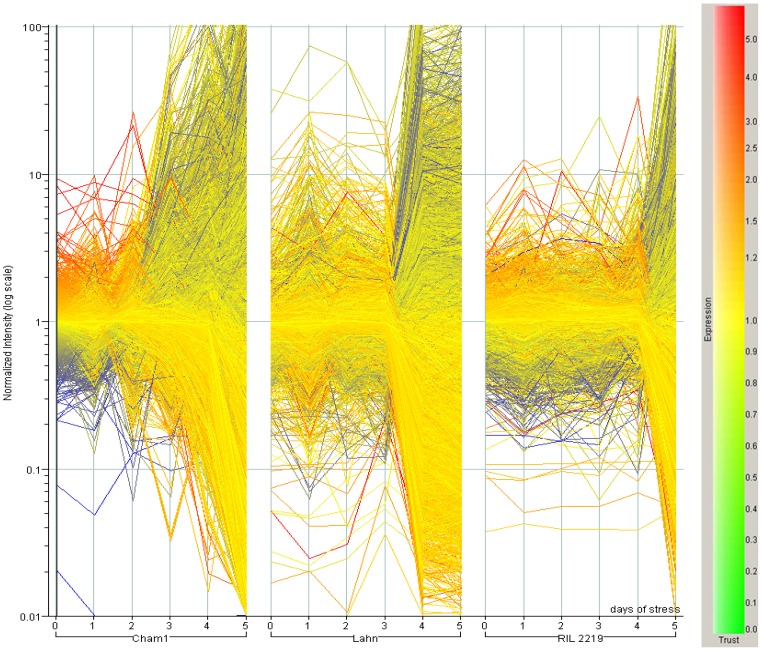
Mean normalised expression of Affymetrix probesets from leaves exposed to water stress. Filtered whole expression dataset of 19062 probes for the three wheat line leaves during the transient of six days of stress analysed using GenespringGX 8.0. Lines are coloured according to their normalised expression for genotype Cham1 at day 0.

Flag leaf RNA samples from three wheat lines subjected to the transient of water stress were submitted for the study of global transcript expression using the wheat Affymetrix gene expression chip. Initial filtering showed that 19062 probes (32% of total probe sets) were modulated in value in response to water stress, genotype, or in combination, in all lines during the stress transient. In the first three days of the stress transient after watering was stopped, probes showed moderate changes in expression and this increased sharply as the stress progressed and leaf RWC declined with some delay shown by RIL 2219 (Figure 2). To analyse probe expression responses as a function of equivalent stress amongst the three wheat lines, this delay was taken into consideration by interpolation of expression values as a function of leaf RWC. ANOVA was then applied to 19062 probes across the three lines and RWC transient, and the results are available for each probe as mean expression values and LSDs for comparisons (File S1). This analysis identified four independent groups showing statistically significant changes with respect to *Line* only (932 probes in File S2), *Stress* only (2579 probes in File S3), *Line*+*Stress* independently (7644 probes in File S4), *Line* × *Stress* interaction (7699 probes in File S5), and a fifth group of 208 probes showing *no statistically significant effect* of either factor (File S6). A detailed analysis of probes annotated to metabolic pathways significantly (p<0.05, LSD) altered from 90-50% leaf RWC for each of the above ANOVA groups was done using the MapMan programme. Some probes annotated to the same gene model and subsequently allocated to more than one of the latter ANOVA groups may be a result of hybridisation to closely related sequences such as gene families, homeoalleles or paralogs.

### Equivalent transcript responses in all lines

**Figure 3:**
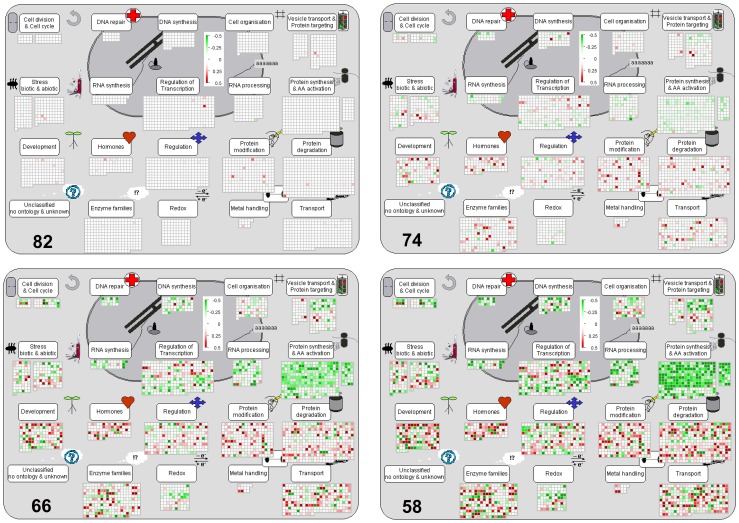
Overview of cell function transcript expression during the water stress transient. Transcript profile changes, at decreasing leaf %RWC (82,74,66,58), were similar across the stress transient for the three wheat lines in probes taken from MapMan bins 15,17,20,21,26,27,28,29,30,31,33,34 in the *Line*+*Stress* ANOVA group dataset (File S4). Results were visualised in MapMan and the colour scale for transcripts that were increased or decreased in abundance were denoted as red and green, respectively.

ANOVA identified probes in *Stress* (File S3), *Line*+*Stress* (File S4) and *Line* × *Stress* (File S5) groups that were statistically altered in expression in response to the water stress transient when compared to controls. Systematic analysis of each of these datasets using MapMan showed similar general trends of metabolic pathway responses to water stress and these are summarized in the following sections with focus on the *Line*+*Stress* (File S4) group as a representative. The 7644 probes in this latter group showed initial differences in expression between the wheat lines at the onset of the stress but this did not influence the profile of response further into the stress transient as RWC decreased; the lines showed similar and parallel stress responses. Probes annotated to specific metabolic pathways (bins) significantly altered from 90-50% leaf RWC are documented in individual spreadsheets in File S4 and Table 1 to enable further mining. Analysis revealed that all represented metabolic pathway bins showed some probes with significant (p<0.05, LSD) over-expression or under-expression of varying orders of magnitude at each leaf RWC (Figure 3). The profiles of the changes in expression of metabolic pathways are complex and varied as leaf RWC decreases, and we focus in this paper on general trends and particularly on changes in regulatory pathways, photosynthetic, primary and secondary metabolism (see following sections).

**Table 1:** Probes with the largest over-expression or under-expression values achieved at the final stress point. Data represents the ratio of change in expression at the final stress level of 50% relative to that at 90 leaf %RWC taken from the ANOVA group dataset stress+line (File S4). Transcript profile changes, as a function of leaf %RWC, were similar across the stress transient for the three wheat lines and probes were annotated to MapMan pathways and bins.

MapMan	MapMan	Affymetrix	MapMan	Expression Change
Bin#	Pathway	Probe	Annotation	50∶90
1	Photosynthesis	ta.3252.1.s1_at	PS2 polypeptide subunits	1.34
2	Sugar metabolism	ta.11114.1.a1_at	ATBETAFRUCT4/VAC-INV (VACUOLAR INVERTASE)	0.93
3	Minor sugars	ta.20649.2.s1_a_at	ATTPS6 (Arabidopsis thaliana trehalose phosphatase/synthase 6)	0.83
4	Glycolysis	taaffx.80151.1.s1_at	PFK2 phosphofructokinase family protein	1.08
5	Fermentation	taaffx.734.1.s1_x_at	ALDH7B4 (ALDEHYDE DEHYDROGENASE 7B4)	0.93
6	Gluconeogenesis	ta.23970.1.a1_at	MASY malate synthase	1.59
7	OPP	ta.9372.1.s1_at	transaldolase	0.57
8	Tricarboxylic acid cycle	ta.2022.2.s1_at	aconitate hydratase, cytoplasmic, putative	0.35
9	Mitochondrial electron transport	taaffx.121853.1.s1_at	AOX1C (alternative oxidase 1C)	0.70
10	Cell wall	taaffx.7177.1.s1_s_at	glycosyl hydrolase family 3 protein	1.91
11	Lipid metabolism	ta.9528.1.a1_at	oxidoreductase, acting on the CH-CH group of donors	0.91
12	N metabolism	ta.1870.1.s1_a_at	GDH2 (GLUTAMATE DEHYDROGENASE 2)	0.78
13	Amino acid metabolism	taaffx.95414.1.s1_at	ATBCAT-3/BCAT3 (BRANCHED-CHAIN AMINOTRANSFERASE 3)	1.50
14	S assimilation	ta.11132.1.s1_at	adenylylsulfate kinase	0.14
15	Metal handling	ta.17991.1.s1_x_at	binding, chelation and storage	0.49
16	Secondary metabolism	ta.11147.1.a1_at	FAH1 (FERULATE-5-HYDROXYLASE 1)	1.11
17	Hormones	taaffx.12175.1.s1_at	ATGA2OX1 (GIBBERELLIN 2-OXIDASE 1)	1.60
18	Cofactors and vitamins	ta.13745.1.s1_a_at	LIP1 (LIPOIC ACID SYNTHASE 1	0.10
19	Tetrapyrrole biosynthesis	ta.11708.1.s1_at	HEMD; uroporphyrinogen-III synthase	0.08
20	Abiotic stress	ta.1316.2.s1_x_at	universal stress proteins (USP)family protein	1.26
20	Stress (biotic and abiotic)	ta.13785.1.s1_at	(CHIB1) acidic endochitinase	1.66
21	Redox	ta.5610.1.s1_at	AHB1 (ARABIDOPSIS HEMOGLOBIN 1)	1.04
22	Polyamine synthesis	ta.3005.1.s1_a_at	ATAIH/EMB1873 (AGMATINE IMINOHYDROLASE)	0.13
23	Nucleotide metabolism	taaffx.97704.1.a1_at	EMB2742 (EMBRYO DEFECTIVE 2742)	0.73
24	Degradation of xenobiotics	ta.8571.1.s1_a_at	lactoylglutathione lyase family protein/glyoxalase I family protein	1.83
25	C1 metabolism	ta.20549.1.s1_x_at	FDH (FORMATE DEHYDROGENASE	0.32
26	Misc enzyme families	ta.11025.1.a1_at	FAD-binding domain-containing protein	1.41
27	RNA processing	ta.26049.1.s1_a_at	MYB4 (myb domain protein 4); transcription factor	1.39
28	DNA synthesis	taaffx.128682.1.s1_at	ENDO4 (ENDONUCLEASE 4)	1.45
29	Protein modification	ta.3507.1.s1_at	SCPL16 (serine carboxypeptidase-like 16)	1.64
30	Signalling	ta.4334.1.s1_at	CPK6 (CALCIUM-DEPENDENT PROTEIN KINASE 6)	1.55
31	Cell cycle, division, vesicle transport and organisation	ta.214.1.s1_at	TUB6 (BETA-6 TUBULIN)	1.37
33	Development	ta.13396.1.s1_at	late embryogenesis abundant	1.87
34	Transport	ta.9295.1.a1_at	PGP21 (P-GLYCOPROTEIN 21); ATPase, coupled to transmembrane	1.42
1	Photosynthesis	ta.22984.2.s1_x_at	LHB1B2 (Photosystem II light harvesting complex gene 1.5); chlorophyll binding	−1.28
2	Sugar metabolism	ta.1197.1.s1_x_at	ATPHS2/PHS2 (ALPHA-GLUCAN PHOSPHORYLASE 2)	−1.04
3	Minor sugars	ta.12952.2.s1_at	pfkB-type carbohydrate kinase family protein	−0.65
4	Glycolysis	ta.9096.2.s1_x_at	ATPPC1 (PHOSPHOENOLPYRUVATE CARBOXYLASE 1)	−0.25
5	Fermentation	ta.28355.2.s1_x_at	ADH2 (ALCOHOL DEHYDROGENASE 2)	−0.52
6	Gluconeogenesis	ta.37.1.s1_at	PMDH2 (PEROXISOMAL NAD-MALATE DEHYDROGENASE 2)	−0.36
7	OPP	ta.29329.1.s1_at	phosphogluconate dehydrogenase	−0.56
8	Tricarboxylic acid cycle	taaffx.123182.1.s1_at	carbonic anhydrase family protein	−1.35
9	Mitochondrial electron transport	ta.1147.3.s1_at	NDH-M (SUBUNIT NDH-M OF NAD(P)H:PLASTOQUINONE DEHYDROGENASE COMPLEX)	−0.56
10	Cell wall	ta.25823.1.a1_at	MUM4 | MUM4 (MUCILAGE-MODIFIED 4)	−1.12
11	Lipid metabolism	ta.4873.1.s1_at	FAD8 (FATTY ACID DESATURASE 8)	−1.32
12	N metabolism	taaffx.12494.1.s1_at	nitrogen regulation family protein	−0.58
13	Amino acid metabolism	ta.23170.1.a1_s_at	DHDPS2 (DIHYDRODIPICOLINATE SYNTHASE)	−1.06
14	S assimilation	ta.19092.1.s1_x_at	SIR (sulfite reductase)	−0.11
15	Metal handling	taaffx.86907.1.s1_at	heavy-metal-associated domain-containing protein / copper chaperone (CCH)-related	−0.13
16	Secondary metabolism	taaffx.12557.1.a1_at	terpenoid metabolism	−1.08
17	Hormones	ta.3526.1.s1_at	NFD5 (NUCLEAR FUSION DEFECTIVE 5)	−0.99
18	Cofactors and vitamins	ta.8003.1.s1_at	UbiE/COQ5 methyltransferase family protein	−0.70
19	Tetrapyrrole biosynthesis	ta.9574.1.s1_at	GUN5 (GENOMES UNCOUPLED 5)	−1.02
20	Abiotic stress	ta.3155.1.s1_at	GFA2 (GAMETOPHYTIC FACTOR 2); heat shock protein binding	−0.80
20	Stress (biotic and abiotic)	ta.27335.1.s1_at	ATMLO1/MLO1 (MILDEW RESISTANCE LOCUS O 1)	−0.84
21	Redox	taaffx.12477.1.s1_at	thioredoxin family protein.	−1.20
22	Polyamine synthesis	ta.24743.2.s1_a_at	SPDS1 (SPERMIDINE SYNTHASE 1)	−0.15
23	Nucleotide metabolism	ta.3179.1.s1_x_at	thymidylate kinase family protein	−1.20
24	Degradation of xenobiotics	ta.7031.3.s1_a_at	GLX2-2 (GLYOXALASE 2-2)	−0.10
25	C1 metabolism	ta.4843.1.a1_at	dihydropterin pyrophosphokinase	−0.73
26	Misc enzyme families	ta.385.2.s1_at	ATPRMT11/PRMT11 (ARABIDOPSIS ARGININE METHYLTRANSFERASE 11)	−1.20
27	RNA processing	ta.26907.1.s1_at	ribonuclease T2 family protein	−1.20
28	DNA synthesis	ta.13372.1.s1_at	hydrolase, alpha/beta fold family protein	−1.06
29	Protein modification	ta.19412.1.s1_at	ribosomal protein L20 family protein	−1.13
30	Signalling	ta.7378.4.s1_at	ATARCA (Arabidopsis thaliana Homolog of the Tobacco ArcA); nucleotide binding	−1.19
31	Cell cycle, division, vesicle transport and organisation	ta.14519.1.s1_x_at	regulators of chromosome condensation (RCC1family).	−0.89
33	Development	ta.7378.4.s1_at	ATARCA (Arabidopsis thaliana Homolog of the Tobacco ArcA); nucleotide binding	−1.19
34	Transport	taaffx.8804.2.s1_at	PIP2;5/PIP2D (plasma membrane intrinsic protein 2;5); water channel	−1.21

#### Early changes

The experimental design uncovered pathways specifically altered early in the stress response with an average of 26% of annotated probes showing changed expression when leaf RWC decreased from 90 to 82% (File S4 spreadsheet summary of early changes). Of those, 247 probes annotated across most of the metabolic bins showed a reduction in expression (File S4 early changes underexpressed spreadsheet) and an even larger number of probes (624) increased in expression with bias towards RNA and protein processing, signalling and transport (File S4 early changes overexpressed spreadsheet). Results also showed that 73% of all annotated probes showed changes in expression as the leaf RWC declined to 74% therefore defining a transition to the next stage of stress response.

#### Regulatory pathways

**Figure 4:**
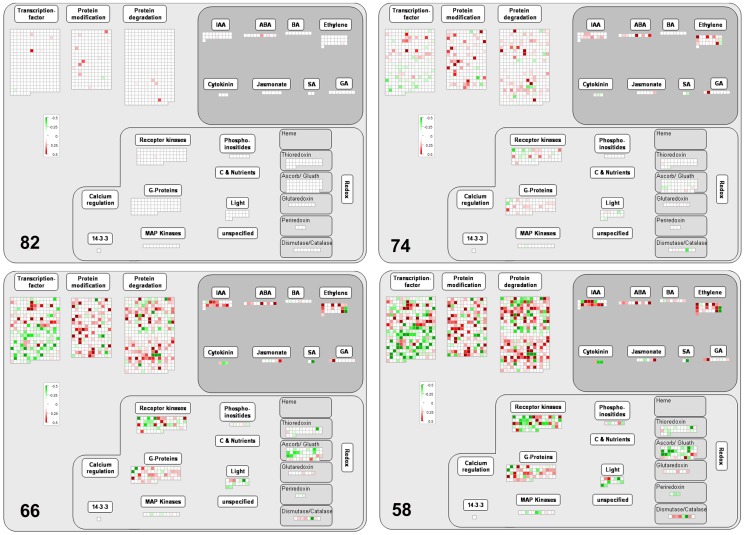
Expression of genes involved in regulatory pathways during water stress. Response of transcripts annotated to MapMan bins 17, 21, 27, 29 and 30 at decreasing leaf %RWC during the water stress transient. ANOVA Dataset and visualisation as for Figure 3.

**Figure 5:**
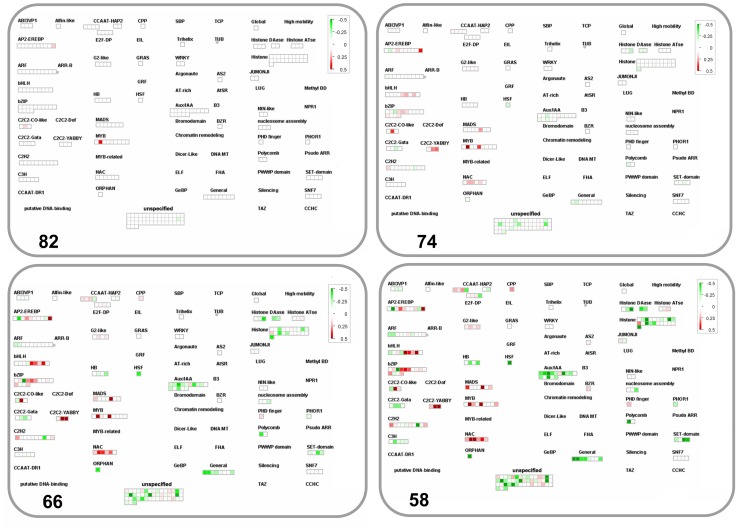
Changes in RNA regulation with focus on transcription during water stress. Expression of probes annotated to transcription from MapMan bin 27 and 28 at decreasing leaf %RWC during the water stress transient. ANOVA Dataset and visualisation as for Figure 3.

The largest number of probes affected by the stress transient were annotated to regulatory pathways with several members showing changes at the very early stage of the stress (82%leaf RWC) (Figure 4). Across the time course of the stress transient, probes annotated to DNA repair and synthesis (chromatin structure: histone, retrotransposon/transposase.hat-like transposase) were identified as significantly altered compared to un-stressed controls with the majority showing under-expression (File S4 bin 28). RNA processing (ribonucleases, RNA helicase, siRNA methyltransferase, splicing), regulation of transcription (transcription factor families or TFs) and RNA binding were particularly affected (File S4 bin 27). Probes annotated to several TFs showed modulation during the whole stress transient (Figure 5), and members belonging to AP2-EREBP, ARF, bHLH, bZIP, C2C2, MADS, Histone ATase, NAC, SET families and MYB showed changes at the earliest stage of stress at 82% leaf RWC with a MYB4 transcript (ta.26049.1.s1_a_at) giving one of the highest measured increases in expression (Figure 5). At 74% leaf RWC, 78% of TF transcripts showed changes in expression profiles and by the final stress point of 50% leaf RWC, 13 showed small changes specific only to that stress point and belonged to ARF, ALFIN, HB MADS, WRKY and histone HDA5 families (Figure 5, File S4 bin 27).

A large number of transcripts in protein modification specifically protein assembly and cofactor ligation, protein degradation (cysteine protease, aspartate protease, autophagy, serine protease, metalloprotease, AAA type, ubiquitin, ubiquitin E1/E2/E3/ubiquitin ubiquitin/ubiquitin ubiquitin protease/ubiquitin proteasome), protein folding, protein glycosylation, post-translational modification and protein targeting were also altered during the stress transient (Figure 4, and File S4 bin 29) with amino acid activation and protein synthesis transcripts showing predominant down-regulation (Figure 3). Transcripts for signalling components, especially a large number of calcium- and G-protein-related and various receptor kinases (leucine rich repeats classes II\ III\VI\VIII-1\X\XI\VIX, DUF 26, legume-lectin, *Catharanthus roseus*-like RLK1, wheat LRK-10 like, proline extension like, wall associated kinase, S-locus glycoprotein like) and others included members belonging to 14-3-3 proteins, sugar and nutrient physiology, light, lipids, MAP kinases, lysine motif and phosphinositides were particularly altered during the stress (Figure 4, File S4 bin 30). These were accompanied by changes in expression of transcripts annotated to redox control; ascorbate and glutathione, dismutases and catalases, glutaredoxins, peroxiredoxin and thioredoxin with most changes measured at 66% leaf RWC (Figure 4, File S4 bin 21).

#### Hormones and stress-specific responses

Probes belonging to each of the major categories of hormone metabolism were modulated in expression as a result of water stress with the majority showing over-expression (Figure 4, File S4 bin 17). They showed close to average change (25%) at the early stress point of 82% RWC except transcripts of abscisic acid (ABA) where 60% of probes showed early phase changes. Nine out of ten probes annotated to ABA metabolism showed over-expression across the transient with the largest change annotated to signal transduction DNA binding transcription activity ABA-responsive element-binding factor (3ABF3/DPBF5) (File S4 bin 17). At 74% leaf RWC most probes annotated to auxin, ethylene, jasmonic acid, salicylic acid and gibberellin metabolism showed modulation in expression and a probe annotated to gibberellin 2-beta-dioxygenase (ATGA2OX1) gave the largest increase amongst all the hormone classes measured at the end of the stress transient (Figure 4, File S4 bin 17; Table 1). Water stress also modulated the expression of a group of probes designated to biotic and abiotic stress-specific responses with 29% of all probes changed at 82% leaf RWC (File S4 bin 20 stress). This included universal stress proteins, genes that respond to cold, heat, touch, salt/drought stress (dehydration-responsive protein-related, drought-responsive family protein, early-responsive to dehydration protein-related/ERD protein-related, DREPP plasma membrane polypeptide family protein, EARLY-RESPONSIVE TO DEHYDRATION 3,4 (ERD3,4)) and a large number annotated to heat shock proteins (HSP: 17, 18, 22, 21, 23, 70, 81, 83, 88, 91, 98, 101, DNAJ, GFA2 and MTHSC70-1).

#### Photosynthesis and energy

**Figure 6:**
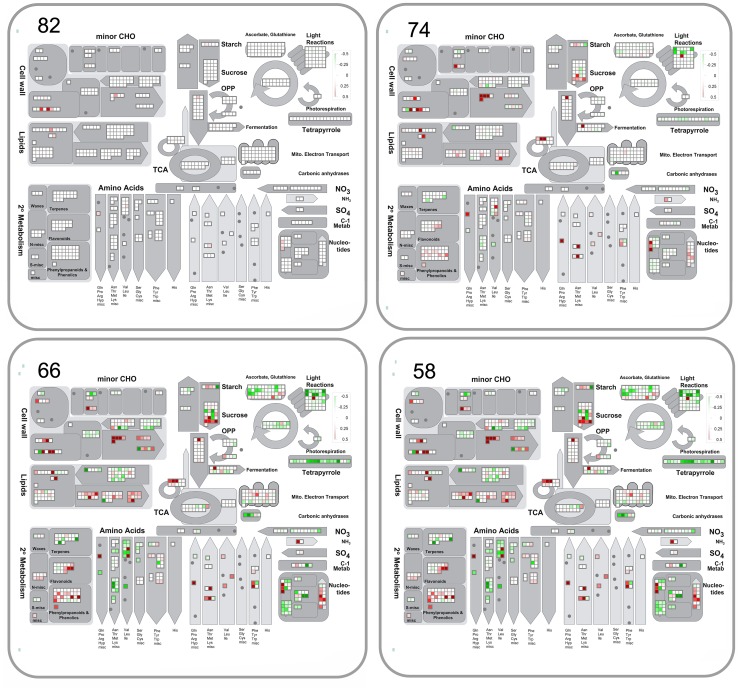
Overview of transcription of genes annotated to primary metabolism during water stress. Expression of probes from MapMan bins 1, 2, 3, 4, 5, 6, 7, 8, 9, 10, 11, 12, 13, 14, 16, 19, 21, 23 and 25, 30 at decreasing leaf %RWC during the water stress transient. ANOVA Dataset and visualisation as for Figure 3.

The majority of probes annotated to photosynthesis were down-regulated during the stress transient with transcripts annotated to LHCII, PS1 and ATP synthase changing early at 82% leaf RWC (Figure 6, File S4 bin 1). Most other transcripts showed changes at 74% leaf RWC, they were annotated to light reactions (LHCII, PS2 and PS1 polypeptide subunits), Rubisco small and large subunit, plastoquinone dehydrogenase complex, ATP synthase beta chain, ferredoxin 3 (ATFD3), serine hydroxymethyltransferase (SHM4), cyclic electron flow (NDHH), phosphoglycerate kinase 1 (PGK1), triosephosphate isomerase (TIM) and phosphoribulokinase (PRK). A few were up-regulated and belonged to phosphoribulokinase (PGK), ATP synthase (VHA-A), subunit of ATP synthase (ATPA), cyclic electron flow (PIFI, NAD2.2) and PS2 polypeptide subunits. This predominant down-regulation of expression of photosynthetic transcripts was accompanied by a reduction in the expression of several probes annotated to tetrapyrrole biosynthesis (Figure 6, File S4 bin 19). Water stress also modulated the expression of transcripts of several enzymes annotated to glycolysis such as pyruvate kinase, phosphofructokinases, phosphopyruvate hydratase (LOS2), and gave increases specifically in phosphoenolpyruvate carboxylase (ATPPC3), fructose-2,6-bisphosphate 2-phosphatase (F2KP) and fructose-bisphosphate aldolase; with phosphofructokinase (PFK2) transcript showing the highest increase measured through the whole transient. Meanwhile phosphoenolpyruvate carboxylase 1 (ATPPC1), pyrophosphate-fructose-6-P phosphotransferase, triose-phosphate isomerase (ATCTIMC) and glyceraldehyde-3-phosphate dehydrogenase (GAPC1, 2) declined (File S4 bin 4). Changes in transcripts of the tricarboxylic acid cycle occurred mainly at 74% leaf RWC, with large changes in carbonic anhydrase (File S4 bin 8) and specific increases in probes annotated to gluconeogenesis at 82%leaf RWC: malate synthase, citrate synthase, malate dehydrogenase PMDH1 and pyruvate dikinase (PPDK) (File S4 bin 6). Various transcripts of mitochondrial electron transport also showed changes, such as ATP synthesis complex I and IV, cyt C and F1-ATPase, with specific increases in alternative oxidase (File S4 bin 9).

#### Sugar, amino acid, lipid and secondary metabolism

Water stress increased the expression of probes annotated to sucrose degradation (sucrose synthase SUS3, 4; fructokinase pfkB-type; hexokinase HXK1), some sucrose invertases (cell wall, neutral and vacuolar) and starch degradation (beta-amylase BMY8/BAM3 and alpha amylase ATAMY1). This trend was accompanied by large decreases in the expression of probes annotated to alpha-glucan phosphorylase 2, cell wall invertases such as beta-fructofuranosidase (ATFRUCT3,5), starch synthesis (synthase, branching, transporter and D-fructose-1,6-bisphosphate 1-phosphohydrolase) (Figure 6, Figure S1, File S4 bin2) and by changes in minor sugar metabolism (File S4 bin3). Various probes annotated to cell wall metabolism, including degradation and synthesis, showed large levels of change especially early in the stress at small water deficit of 82% RWC (Figure 6, File S4 bin10). Water stress produced major modulations in expression of transcripts of amino acid metabolism in both synthesis and degradation: there were decreases in the synthesis of arginine, histidine, threonine, lysine, leucine, cysteine, methionine, aspartate, asparagine, branched chain amino acids, serine, tryptophan, precursors for aromatic amino acids. There were however increases in transcripts for the synthesis of cysteine, methionine, glutamate decarboxylase 1, 4-aminobutyrate transaminase (GABA-T), aspartate transaminase, proline synthase (P5CS2), some branched-chain aminotransferase, acetolactate synthase small subunit and various aromatic amino acids. These changes were accompanied by decreases in transcripts for the degradation of histidine, GCN5-related N-acetyltransferase, threonine, methionine, lysine, asparagine, tyrosine and tryptophan and increases in those involved in the degradation of arginine, valine, branched-chain group, methionine, lysine, tyrosine and tryptophan (Figure 6, File S4 bin 13).

**Figure 7:**
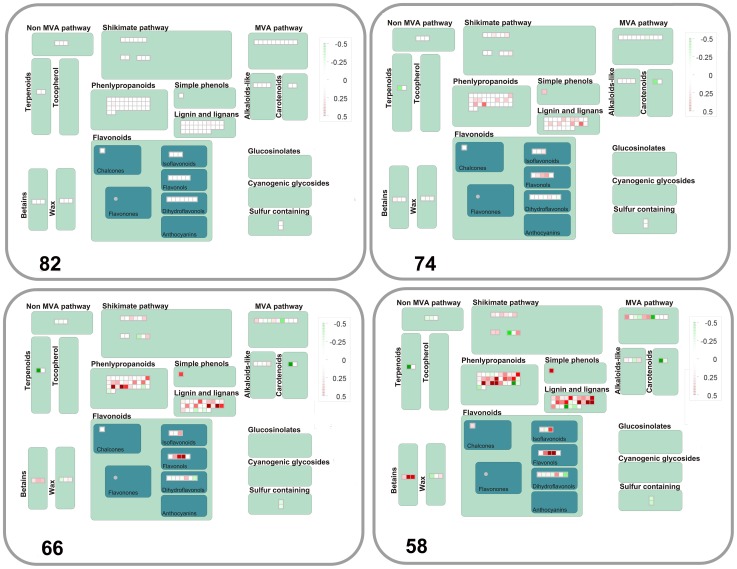
Changes in transcription of genes annotated to secondary metabolism during water stress. Expression of probes annotated to MapMan bin 16 at decreasing leaf %RWC during the water stress transient. ANOVA Dataset and visualisation as for Figure 3.

Water stress caused down-regulation of transcripts of nitrate reductase (NIA2) and increases in those for glutamine synthetase (cytosolic Gln1;4), glutamate dehydrogenase (GDH2) and glutamate synthase (GLT1) (Figure 6, File S4 bin12). Changes in the expression of probes annotated to lipid metabolism were also measured for biosynthetic enzymes of fatty acid desaturation, fatty acid synthesis and elongation, steroids, squalene and sphingolipid metabolism, lipid degradation (beta oxidation, lipases, lysophospholipases) lipid transfer proteins and phospholipid synthesis (Figure 6 and File S4 bin11). Probes were also altered in nucleotide metabolism with overall down-regulation in purine and ATP synthesis, whilst some coding for pyrimidine and CTP synthase were up-regulated (Figure 6, File S4 bin 23). Probes annotated to several secondary metabolic pathways were affected by the stress with predominant increases in expression of phenylpropanoids, lignin biosynthesis, flavanoids, betaine, and wax metabolism (Figure 7, File S4 bin16).

#### Cell organisation, transport and development

Probes annotated to cell cycle, cell division, cell organisation, development and vesicle transport were modulated by water stress (Figure 3, File S4 bin 31, 33) with probes annotated to late embryogenesis abundance showing over-expression at the earlier phases of the stress (File S4 bin 33). A large number of transcripts annotated to transport showed changes in expression such as those belonging to ABC transporters and multidrug resistance proteins; transporters for : amino acids, ammonium, calcium, sugars, sucrose, metals, cyclic nucleotide or calcium regulated channels, peptides and oligopeptides, phosphate, potassium, sulphate and unspecified ions; H+ transporting pyrophosphatase; Major Intrinsic Proteins (PIP, TIP); metabolite transporters at the envelope and mitochondrial membrane; NDP-sugars at the ER; p- and v-ATPases, p- and v-ATPases.H+-transporting two-sector ATPase (File S4 bin 34).

Another group of probes, assigned to miscellaneous enzyme families involved in various metabolic processes, were modulated in response to the stress and included several members of cytochrome P450 which showed relatively large changes in expression (File S4 bin 26). Annotated probes from all metabolic bins sorted by the largest increase in expression measured at the last time point of the stress are summarised in Table 1. Probes contributing to most differences across the whole stress response were identified using PCo, see below.

### Genotype-dependent responses

**Figure 8:**
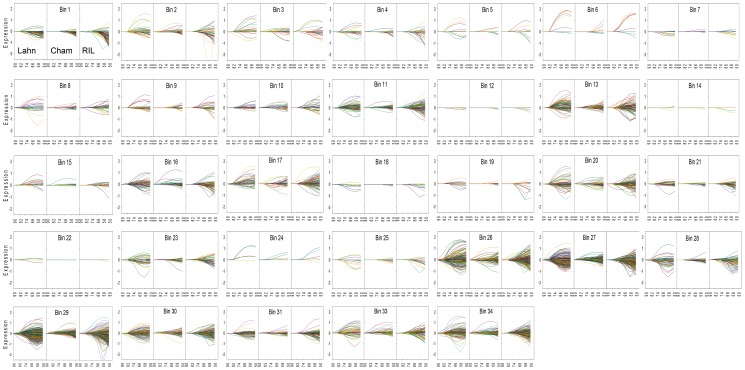
Differences in the global transcription profiles of the three lines under stress. Expression of probes from the three lines, Lahn, Cham1 and RIL2219, as a function of leaf %RWC during the water stress transient, visualised in MapMan for all metabolic bins. For every bin, the same colour probe is plotted for each wheat line to enable direct comparison. Transcript profiles belong to the *Line* × *Stress* ANOVA group dataset (File S5).

The ANOVA group *Line* × *Stress* comprised 7699 probes documenting an interaction between the two independent variables wheat line (two parents and RIL 2219) and stress (transient); probes that showed statistically significant responses to the stress and also measured differences between the lines in the way they responded to the water deficit. The global nature of the stress response, in all three lines, is demonstrated by the many pathways affected and by the large number of affected probes annotated to regulatory pathways presented in File S5 and Figure 8. These were essentially similar to those documented by the other ANOVA groups *Stress* (File S3) and *Line*+*Stress* (File S4) in showing down-regulation of photosynthesis, reconfiguration of primary and secondary metabolism and a predominant transcript modulation of regulatory pathways for RNA and protein modification. Nevertheless, each of the 7699 probes showed a statistically significant difference in expression between the lines in terms of absolute value, starting value and/or pattern of change across the transient. These are documented for each probe and partitioned into MapMan metabolic bins highlighting probes showing changes early in the stress transient for each line (File S5 early changes sorted for each line spreadsheets, Table 2). Inspection of the early phase of the stress at 82% leaf RWC showed the greatest changes in gene expression had occurred in Lahn, with 10% less changing in Cham1 and even 60% less in RIL 2219 with the latter being the most conserved. Graphical plots of the two parent’s transcripts showed distinctly different profile responses to water stress, at the same leaf %RWC, across several metabolic pathways, whilst RIL 2219 combined features derived from either parent and also showed some distinct responses either in the trend or in the magnitude of the transcript expression (Figure 8). Moreover, this plot uncovers a more responsive transcriptome in Lahn, with at least one probe per bin showing a higher expression value than either Cham1 or RIL2219; the only exceptions being for bins 10 (cell wall), 15 (metal handling), 16 (secondary metabolism) and 17 (Hormones).

**Table 2:** Top two probes showing largest changes in expression during the early phase in the stress for each wheat line. Data represents the ratio of change in expression (over and under-expression values) at 82% relative to that at 90 leaf %RWC taken from the ANOVA group dataset *line x stress* (File S5).

MapMan	MapMan	Affymetrix	82∶90
Bin#	Annotation	Probe	Lahn
26	misc.cytochrome P450	ta.3813.1.a1_at	**0.61**
27	RNA.regulation of transcription.Aux/IAA family	ta.10395.1.s1_a_at	**0.47**
34	transport.peptides and oligopeptides	taaffx.65026.1.a1_at	**−0.31**
29	protein.synthesis.ribosomal protein.prokaryotic.mitochondrion.50S subunit.L2	ta.28514.2.s1_at	**−0.46**
			**Cham1**
16	secondary metabolism.phenylpropanoids.lignin biosynthesis.CAD	ta.2659.1.s1_at	**0.38**
16	secondary metabolism.simple phenols	ta.4455.1.a1_at	**0.35**
34	transport.metal	ta.9290.1.s1_at	**−0.11**
20	stress.biotic	taaffx.108556.1.s1_at	**−0.11**
			**RIL2219**
17	hormone metabolism.ABA.synthesis-degradation.9-cis-epoxycarotenoid dioxygenase	taaffx.76007.1.s1_at	**0.46**
29	protein.degradation.ubiquitin.E3.SCF.FBOX	taaffx.12903.1.a1_at	**0.26**
8	TCA/org. transformation.carbonic anhydrases	taaffx.37789.1.a1_at	**−0.11**
20	stress.abiotic.heat. Heat shock protein HSP21	ta.202.1.s1_at	**−0.36**

#### Identifying probes responsible for most differences between lines

**Figure 9:**
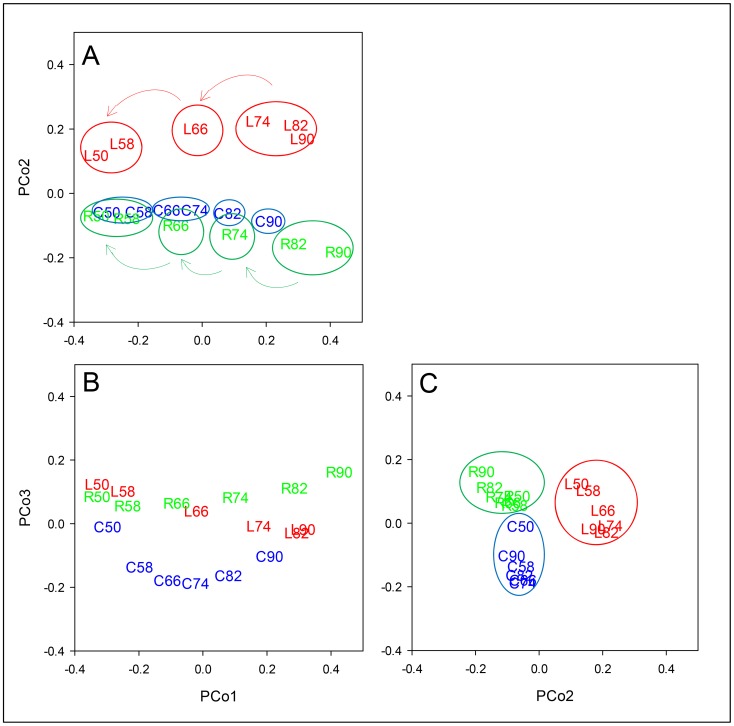
PCo plots for the expression of all 19062 probe dataset. The first three PCos account for 72.8% of the variation represented in the (reduced) similarity matrix for the 18 wheat line by RWC combinations. Visualisation of the combinations in the three dimensions was achieved by plotting PCo1 *vs.* PCo2 (A), PCo1 *vs.* PCo3 (B) and PCo2 *vs.* PCo3 (C). Letters L, C and R represent the lines Lahn, Cham1 and RIL 2219 respectively at 90-50 leaf %RWC values. Arrows on plot A show the direction of the stress transient of decreasing leaf RWC and the free-drawn circles around the points highlight potential functional states for each line, inferred from distance between points.

To explore patterns and sources of variation responsible for differences between wheat lines from those due to the stress transient, the whole RWC-interpolated dataset of 19062 probes was submitted to PCo analysis. The analysis identified three major PCos and revealed clear zoning of each line across the stress transient and also between the lines with little evidence of randomness, demonstrating the structure, global nature and tight regulation of the response to water stress (Figure 9). PCo1 accounted for 47.84% of the total variation in the sample-to-sample similarities and separated the samples with regards to increasing stress for all three lines (Figure 9 A, B). The plot suggests that each line transcriptome transitions smoothly across the stress in the direction of the arrows and the circles highlight possible system functional states Figure 9 A. PCo2, explaining 15.01% of the variance, separated the data as regards line-to-line differences, in particular separating Lahn from Cham1 and RIL 2219, these latter two lines showing more similar coordinates on this axis (Figure 9 A, C). PCo3, accounting for 9.95% of the variance, also pulled out line-to-line differences, but this time extracting differences for Cham1 from the other two lines (Figure 9 B, C). PCo2 and PCo3 therefore uncovered the global ways in which each of the two parents Cham1 (PCo2) and Lahn (PCo3) are similar to, or different from, the offspring, RIL 2219 which in turn combines transcriptome patterns derived from both parents in terms of its response to decreased RWC. The plot of PCo1 *vs.* PCo2 shows that RIL 2219 and Cham1 are closer, or more similar, throughout the transient, and that all lines become more similar as the stress progresses to the severe point at 50 leaf % RWC (Figure 9 A). Furthermore, the plot of PCo1 *vs.* PCo3 (Figure 9 B) showed that the response of Cham1 differed from the other two lines as RWC fell from 82 to 58%. Plotting PCo2 *vs.* PCo3 (Figure 9C) revealed that PCo3 can be used to consider relative within-line stability as the stress increases, three distinct line-specific circle clouds of points are shown, the most disparate set being for Lahn (more susceptible parent) and the most clustered set being for Cham1 (more resistant parent) apart from its point at 50% RWC, which is a considerable distance away from the others. The set of points for the RIL 2219 is more similar to that of Cham1 than to that of Lahn, viewed from the PCo2 axis. Therefore, it is clear that PCo2 and PCo3 pull out differences between lines at all stress points including the relatively stress-free condition of 90% RWC. Importantly, the spread of coordinates of samples at 90% leaf RWC for all three lines demonstrates the intrinsic differences that exist between the transcriptomes of each line before the onset of water deficit.

**Figure 10:**
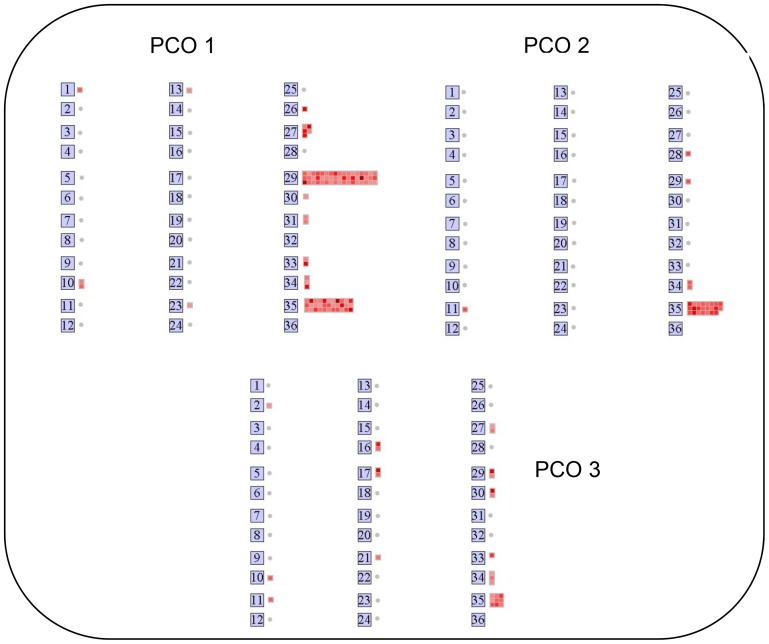
MapMan visualisation of the top PCo probes responsible for differences. 5% of probes with greatest F-values following PCO (data from File S7) showed differences in distribution across all metabolic bins for PCo1, PCo2, and PCo3. For presentation in Mapman, FPCO maximum values were set at 2000, 700 and 450 for PCo1, PCo2, and PCo3 respectively.

The analysis also enabled the identification of the major probes responsible for differences between lines and those due to the stress transient. The 5% of probes most significantly correlated (F-tests) with the PCo coordinates PCo1, PCo2 and PCo3 (File S7) were imported into the MapMan programme for assignment to bins and results showed differences in the distribution of these probes across the metabolic pathways (Figure 10). Thus, PCo1 identified 1–3 probes in annotated bins 1 (photosynthesis), 10 (cell wall), 13 (amino acid metabolism), 23 (nucleotide synthesis), 26 (misc. enzyme families), 30 (signalling), 31 (cell organisation), 33 (development), 34 (transport), and several belonging to regulatory bins 27 (RNA processing), 29 (protein modification) and bin 34 (transport). The probe with the greatest F-value (Ta.3659.1.S1_a_at) gave no annotation using MapMan, so we explored this further using PLEXdb to obtain a putative annotation to heat shock factor-binding protein 1, using rice alignment at Gramene (LOC_Os09g20830.2). PCo2 identified probes in annotated bins 11 (lipid metabolism), 28 (DNA), 29 (protein modification) and 34 (transport); the probe with the highest F-value (taaffx.3401.1.s1_at) was annotated to vacuolar sorting protein 39, having small GTPase regulator activity and association with the TGF beta receptor. Finally, PCo3 identified probes in bins 2 (major sugars), 10 (cell wall), 11 (lipid metabolism), 16 (secondary metabolism), 17 (hormones), 21(redox), 27 (RNA regulation), 29 (protein modification), 30(signalling) and 34 (transport) with the most influential probe ta.16038.1.s1_at annotated to hormone metabolism- ABA inducible, encoding one of five HVA22 homologs in *Arabidopsis*; an ABA- and stress-inducible gene.

Another source of variation that can influence the expression values for probes between the three lines is allelic variation at the gene sequence level. This may affect the binding of a particular probe from a given probe set and the extent of its occurrence in our study was estimated by calculating the ratio of a probe signal to that averaged over the eleven probes in the corresponding probe set. A preliminary analysis was done for probe sets annotated to transcription factors, bin27, for the three ANOVA groups, *line*, *line+stress* and *line x stress* where such differential hybridisation may be an important consideration when comparing expression differences between the lines. The results showed that for the *line only* ANOVA group, 15 probes out of 396 probes (36 probe sets each containing 11 individual probes) showed some evidence of differential hybridisation between the lines but with no stress response (File S8). The analysis also showed that for the *line+stress* ANOVA group, 140 individual probes out of a total of 4862 showed statistically different hybridisation when compared to the average for probe sets; however, this component did not affect the response to water stress since all lines behaved similarly. The group for which such allelic hybridisation may contribute to the differential stress response amongst the three lines is the *line x stress* ANOVA group and results showed that 108 individual probes out of a total of 4807 (File S8) may have such an interaction; further verification in new studies would be required to confirm this.

### Probes showing no response to stress

The ANOVA group, *Line*, identified 932 probes that were significantly different in transcript expression between the three lines but did not show changes in response to the water stress transient (File S2). We also identified 208 probes (File S6) that showed no statistical difference either between lines or in response to the stress but which were selected during the initial filtering step using Genespring GX. These two ANOVA groups could be used to provide potential control genes for future water stress transcript expression studies in durum wheat.

## Discussion

### Durum wheat gene expression responds to water stress in a global and regulated manner

The stress transient enabled the capture of the complexity of the plant’s response to water stress as a first step in dissecting inherent features of the biological system under perturbation. The mathematical models identified all statistically significant responses, without filtering, which uncovered all changes in transcript expression, not just focussing on a particular arbitrary magnitude of change, or exploring only specific pathways; unlike most published studies. The results showed that the durum wheat transcriptome responded in a global manner to progressive water stress as demonstrated by the modulation of a large number of probes annotated to various metabolic pathways in all three lines; this has been observed in previous datasets on various species [36], [37], [38], [39], [22], [40], [41]. Our study also showed evidence of a highly regulated response in the proportionally large number of probes coding for regulatory genes (DNA, RNA and protein modification, transcription factors, hormones and signalling) specifically altering expression at the early phases of the stress. This supports and demonstrates the fundamental components of regulatory networks of sensing, transducing and responding that operate at the genome level when exposed to abiotic stress [16], [42]. These are essential features of system robustness that enable the maintenance of function under stress through immediate changes in some metabolic pathways, documented in File S3, File S4, File S5, and also via the launch of downstream pathways such as protective secondary metabolism. Particular attention has been given to transcription factors as major control points in the plant system’s response to water stress [43] and here we present the first overview of the water stress response profile of several durum wheat family members changing expression over the range of RWC. We identified particular groups of probes annotated to AP2-EREBP, ARF, bHLH, bZIP, C2C2, MADS, Histone ATase, SET and MYB4 families that were altered in leaf transcription early on in the stress transient when no other physiological changes were measurable. This reinforces the importance of transcription factors in the regulation and coordination of transcriptional and metabolic responses which form essential components of regulatory networks. Another interesting result is that of the modulations in transcripts for heat shock proteins throughout the stress transient. Molecular chaperones play a crucial role in the reestablishment of cellular homeostasis under abiotic stress [44] and alterations to heat shock proteins, caused by shared metabolic responses, are common to abiotic stresses such as water deficit and heat and thus share fundamental mechanisms as discussed by [17].

The changes in expression of a large number of probes annotated to protein modification and signalling in addition to hormones further supports this integrated global view of the wheat genome stress response as the system aims to balance key processes such as protein synthesis, cellular maintenance, metabolism, growth and development. Thus, these multifaceted changes in gene expression of the regulatory pathways are best interpreted as essential features of robustness as the system adjusts to perturbations and moves from one functional state to another, as envisaged in Figure 9A. Therefore, the tendency to consider particularly large increases in gene expression as all candidates for ‘improving’ plant growth under drought is flawed and such individual changes should be understood in the context of robustness with all its elements of degeneracy, feedback, homeostasis and environmental tracking operating within the biological system [45], [46]. To establish how this highly regulated response is co-ordinated at the gene expression level across the stress transient, mathematical and bioinformatics models can be used to uncover new gene networks for water stress response in cereals and other species [47], [42]. Recently, co-expression network algorithms have been successfully used to explore gene networks, network architecture and control hubs, and to sketch out new hypotheses for further testing [48], [49]. Future studies would also enable a comparison of our datasets with similar ones from barley, bread wheat, maize and rice, in order to determine which transcriptome responses are conserved as a common framework amongst cereals and which are species- and environment-specific.

### Water stress downregulates photosynthesis and alters transcripts for sugar metabolism

Durum wheat leaves exhibited a decline in photosynthetic parameters, osmotic adjustment and increases in ABA content as the stress progressed through the transient of declining leaf RWC, as expected [50]. This was accompanied by a lowering of photosynthetic, photorespiratory and tetrapyrrole biosynthetic transcripts which reflects a down-regulation of light capture and carbon fixation. Such patterns have been seen in previous studies in dicots, rice, barley and wheat and support a common framework for the plant’s photosynthetic responses to water stress [48]. We also found that the lowered expression of photosynthetic transcripts was coupled by increases in expression for sucrose and starch breakdown, and a decrease for starch synthesis. This implies a requirement for soluble sugar availability for respiration, solute homeostasis and/or signalling throughout the plant [51]. The perturbations observed in key regulators of carbon metabolism and glycolysis such as phosphofructokinase, phosphoenolpyrovate carboxylase and pyruvate kinase with a concomitant increase in expression of enzymes of gluconeogenesis and sucrose invertases may also contribute to an increase in sucrose hydrolysis [52] in agreement with analysis of metabolites under water deficit [51]. These results provide some evidence for leaf osmotic adjustment as a strategy to maintain cell turgor pressure [51], [53], it might also reflect the need for respiratory substrates as respiration is maintained when photosynthesis slows and ceases.

### Water stress reconfigures transcription of primary and secondary metabolic pathways

The complex changes measured in transcripts coding for amino acid and lipid metabolism, GABA-T, and various aminotransferases, demonstrate the reconfiguration of primary metabolism under stress as the system moves between different metabolic ‘states of function’. The overall downregulation of transcripts for synthesis of most amino acid groups and an increase in the expression of transcripts involved in amino acid breakdown occurred downstream in the stress response as the plant adjusts metabolism from synthesis towards remobilisation and protective metabolic pathways, in addition to providing substrate for respiration as photosynthesis declines. This is demonstrated by specific changes in transcripts for key enzymes involved in amino acid and inorganic nitrogen assimilation. For example, the down-regulation of nitrate reductase implies a decreased assimilation of inorganic nitrogen and this was accompanied by over-expression of transcript annotated to cytosolic glutamine synthetase, glutamate dehydrogenase and glutamate synthase which in turn would release glutamate and 2 oxoglutarate for recycling, thus accelerating remobilisation and senescence [54], and possibly increasing the production of proline via glutamate. This enables increased remobilisation of nitrogen assimilates as metabolism is adjusting the decline in growth with primary synthesis under stress conditions.

We also measured changes in transcripts coding for secondary metabolism such as phenylpropanoids, lignin biosynthesis, flavonoids, betaine, and wax metabolism, thus providing evidence for a protective adjustment role in the stress response. The increases we observed in the expression of an orthologue for proline biosynthesis, P5CS2 might be considered as providing proline for osmotic adjustment and/or reflecting an imbalance in amino acid regulation. Other adaptive responses such as changes in expression of transcripts for cell wall biosynthesis, measured early on in the stress at small changes in RWC, demonstrate the dynamism of mature cell walls in response to decreased turgor and supports recent studies [55], [56], [57], [11]. We also observed that a range of responsive transcripts to both biotic and abiotic stress were also modulated and this supports the existence of common global gene networks for environmental stress responses; interestingly [35] also noted similar changes in biotic pathogen-related gene expression under water stress in wheat.

### Wheat lines show constitutive differences in gene expression

The time series approach allowed the dissection of the complex transcriptional response into the wheat specific component and those specific to changes in RWC, as well as identifying probes showing an interaction between the two. A large number of probes showed inherent or constitutive differences in transcript expression between the wheat lines before the stress had started; all probes in File S2, File S4 and some probes in File S5. This is clearly seen in the PCO plots in Figure 9 where the values at leaf 90%RWC are dispersed across all axes for all three lines. Large systemic differences in gene expression between germplasm within a species, including between siblings and parent, have been shown to be strikingly common in rice [58], emmer wheat [41] and Arabidopsis [59]. It is unclear however, at this stage of study, how these differences contribute towards the ability of a line to resist stress and these results should be coupled to expression QTL studies dissecting GxE to enable further evaluation. Studying the transcriptome of wheat lines under both controlled and field conditions would then enable us to understand the importance of these global differences between lines in terms of the variation in the final integrated responses to the stress in the complex field environment and we are currently evaluating such datasets from field trials. Integrating genomic high-throughput datasets with fine mapped QTLs in future studies would consequently bridge the link between changes at the molecular level with downstream yield-related traits underpinning final grain yield [21].

### Differential gene expression responses amongst wheat lines under stress

The three wheat lines responded to water stress in a global framework of common changes of gene expression in regulatory, primary, secondary, and protective metabolic pathways. In addition, we documented thousands of probes that showed significant differences in the transcript response for each wheat line during the stress transient (File S5). These differences were analysed at equivalent leaf RWC reflecting real differences in gene responses between the susceptible and resistant lines which may prove to be the most interesting in terms of targets for genotype-specific responses under water stress. The probe responsible for inducing most variation across the whole dataset in terms of line-to-line stability, derived from PCo3, was annotated to an ABA inducible HVA22E or a late embryogenesis abundant protein which is implicated in a protective stress response by regulating vesicular traffic in stressed cells [60]. This, and other probes identified by PCo, can now be tested in new mechanistic studies to establish whether they are new regulatory control points for drought resistance in durum wheat. An interesting result from our study was that Lahn, the drought sensitive parental line, had a higher number of modulated probes in response to the stress, especially at the initial phase of the transient. This was true at equivalent leaf %RWC, across most pathways, and in comparison to the other two lines, especially RIL2219 which had the lowest number of modulated probes at that stage. An examination of the literature highlighted few comparative analyses in rice [39], a maize drought resistant RIL line with fewer modulated genes under stress [61] whilst drought in maize landraces showed a mixed picture [62]. Therefore, it may also be important to consider the metrics of gene expression changes in addition to the nature of the genes and the networks involved.

In addition to differential gene expression documented between the three lines, we have estimated the contribution of allelic differential hybridisation to the transcripome response. We have identified a small proportion of such individual probes in bins annotated to transcription factor probe sets, most of which did not influence the response of probes to water stress (ANOVA group *line only* and *Line+stress*). Nevertheless, a handful were identified as promising in the ANOVA group *Line x stress* and one in particular, probe # 602715 belonging to the probe set ta.28513.1.s1_s_at and annotated as an Auxin Response Factor family, deserves close verification since it was also identified by PCo analysis as a major candidate for within-line stability under stress (File S7 and S8). These represent possible candidates for marker development and should be further examined in future studies to verify their potential for durum wheat marker assisted breeding.

One of the most striking findings is the co-ordinated and highly structured transcriptome response for each wheat line across the water stress transient as demonstrated by the lack of scatter and high level of structure in Fig. 9C. Each line started at a different point along the axes for the three PCos demonstrating inherent differences in transcript expression across most metabolic pathways and this distinctness is maintained during the transient with some indication of reaching closer values at the final stress point. This provides strong evidence for a distinct cultivar-specific global transcriptome response in each wheat line so that the whole genome is responding in a highly coordinated manner. Very few studies have undertaken such a global analysis and for those that have utilised PCo, a similar interpretation can also be derived [22]. These, together with constitutive differences are important findings and should be factored into current simplistic efforts to identify a singular mechanism for drought resistance and genetic modification of a particular selected metabolic pathway. We argue that a shift in thinking is required to integrate high throughput datasets to advance our understanding of plant responses under stress and that robustness theory may offer such a framework.

### Physiological traits differ before and during the stress

The most drought resistant RIL 2219 line had higher photosynthetic and transpiration rates, greater stomatal conductance and ABA content prior to the initiation of stress and was also able to maintain greater RWC and leaf water potential for a longer period than either parent. It is interesting to note that parent Cham1 has relatively larger stomatal conductance when compared to other breeding varieties [63]. The greater rate of leaf transpiration may seem counterintuitive for a drought resistant line, but if coupled with lower leaf area and/or bigger capacity to capture water (for example from a more efficient root system) then it can afford to transpire slightly more and maintain its stomata open for longer to allow entry of CO_2_ for fixation. We argue that this could provide an important advantage in the sustained accumulation of assimilate, especially during the fast vegetative growth period where water may be available, and hence increased grain yield if integrated over the whole growth season. In addition, the higher leaf transpiration rate for RIL2219 may also reduce the canopy temperature, this being another added benefit for durum wheat during terminal stress when drought is often compounded with heat stress [64], [65], [66]. We propose that these combined factors are responsible for RIL2219’s inherent greater capacity for carbon assimilation and thus the ability to maintain higher yield stability than either parent under drought in the field, as supported by datasets from a large number of field trials under various drought gradients (unpublished observations). Evidence exists that wheat breeders have inadvertently selected for higher stomatal conductance in their quest for greater yield potential under optimal conditions, and this has also proved to be beneficial under water-limiting Mediterranean environments [67], [64], [68], [69], [70], [65], [71], [72], [73], [74]. There was also evidence for greater osmotic adjustment ability in the two drought resistant lines Cham1 and RIL 2219 than in Lahn. Taken together, improving osmotic adjustment, and increasing carbon fixation and stomatal conductance may define one physiological strategy for success in breeding high yield stability wheat for Mediterranean drought prone environments. Further systems studies are now necessary to link the transcriptome, proteome, metabolome and phenome of wheat to establish how the different levels of function interact to produce a final yield under stress.

## Conclusions

We found evidence of a common global framework of physiological, biochemical and gene expression responses in all three wheat lines under water stress. The stress transient identified the predominant modulation of the transcriptome of regulatory pathways early in the stress response, downregulation of photosynthesis, reconfiguration of primary metabolism and the launch of protective downstream pathways at various leaf RWC of relevance to terminal drought experienced by crops under field conditions. We also uncovered genotype-specific transcriptome and physiological differences before and during the stress amongst the three wheat lines which suggests that each wheat line functions within a genome-specific structure that form the basis of different stress-resistance strategies. We interpret the results by ascribing systems–based concepts of robustness as a fundamental framework of the plant’s response to perturbation under stress. By targeting our studies to durum wheat lines from the breeding programme at ICARDA, our results should be useful to breeders searching for novel variation and new concepts to test and exploit in wheat cultivar improvement.

## Supporting Information

Figure S1 **Expression of genes in sugar metabolism during stress.** Expression of probes annotated to MapMan bin 2 as a function of decreasing leaf %RWC. ANOVA Dataset and visualisation as for Figure 3.
(TIF)

File S1 **ANOVA results of probe expression values of three wheat lines in response to water stress.** Whole dataset of 19062 probes interpolated values, pre-ANOVA, for individual replicates at each leaf %RWC level and for the three lines Lahn, Cham1 and RIL2219 in spreadsheet ‘Interpolated data at RWC levels’. ANOVA, log (base2), split the whole dataset into 5 independent statistical groups represented by individual spreadsheets: probes showing differences in expression between the lines but not during the stress in spreadsheet ‘line only 932 probes’; probes showing differences in expression as a result of the stress transient but not between lines in spreadsheet ‘stress only 2579 probes’; probes with differences in expression between the lines at the onset of the stress with the profile changing in the same way during the stress transient in spreadsheet ‘line+stress 7644 probes’; probes with differences in expression due to line and stress combined, showing an interaction in spreadsheet ‘line x stress 7699 probes’; and probes with no differences due to stress or line after initial filtering in spreadsheet ‘no effect 208 probes’. Statistical results are given as least significant difference (LSD) at 5% and 1% levels of significance for each of the ANOVA groups.
(XLSX)

File S2 **MapMan analysis of ANOVA group** ***line only*** **932 probes.** Probes showing statistically different expression values between the three lines Lahn, Cham1 and RIL2219 but with no effect of the water stress. Data were calculated as expression value beyond the relevant LSD (5%) given in File S1 when comparing means for each probe between the three lines Lahn, Cham1 and RIL2219, or otherwise zero when there was no significant difference. Probes are presented by Affymetrix id and MapMan annotation to bin name, pathway and description of gene model.
(XLSX)

File S3 **MapMan analysis of ANOVA group** ***stress only*** **2579 probes.** Probes showing no differences in expression between the three lines Lahn, Cham1 and RIL2219 but changing in response to the stress. Data were calculated as expression value beyond the relevant LSD (5%) given in File S1 when comparing means for stressed (82, 74, 66, 58 and 50% leaf RWC) to control (90% leaf RWC) conditions for each probe, or otherwise zero when there was no significant difference. Probes are presented by Affymetrix id and MapMan annotation to bin name, pathway and description of gene model. Analysis of results are presented in individual spreadsheets for all MapMan bins and sorted for different leaf %RWC. In addition, probes with the greatest levels of over-expression or under-expression measured at the earliest stress point of 82% are highlighted in spreadsheets ‘early changes over-expression’ and ‘early changes under-expression’, respectively.
(XLSX)

File S4 **MapMan analysis of ANOVA group** ***line+stress*** **7644 probes.** Probes showing consistent differences in expression between the three lines Lahn, Cham1 and RIL2219 in response to the stress transient –*i.e.* they show a parallel profile of change in transcript response across the stress transient. Data were calculated as expression value beyond the relevant LSD (5%) of data in File S1 when comparing means for stressed (82, 74, 66, 58 and 50% leaf RWC) to control (90% leaf RWC) conditions for each probe, or otherwise zero when there was no significant difference. Data were also calculated as the differences in expression between the three lines. Probes are presented by Affymetrix id and MapMan annotation to bin name, pathway and description of gene model. Analysis of results are presented in individual spreadsheets for all MapMan bins and sorted for different leaf %RWC. In addition, probes with the greatest levels of over-expression or under-expression measured at the earliest stress point of 82% are highlighted in spreadsheets ‘early changes over-expression’ and ‘early changes under-expression’, respectively. Percentage changes in probes at 82∶90 and 74∶90% leaf RWC stress points are also included in spreadsheet ‘summary of early changes’ and calculated as a percentage of probes changed at 82∶90 or 74∶90 as a proportion of all probes showing changes in each specific bin across the whole transient.
(XLSX)

File S5 **MapMan analysis of ANOVA group** ***line x stress*** **7699 probes.** Probes showing differences in expression between the three lines Lahn, Cham1 and RIL2219, *and* in response to the stress; they show an interaction. Data were calculated as expression value beyond the relevant LSD (5%) given in File S1 when comparing means for stressed (82, 74, 66, 58 and 50% leaf RWC) to control (90% leaf RWC) conditions for each probe from each of the three lines Lahn, Cham1 and RIL2219, or otherwise zero when there was no significant difference. Probes are presented by Affymetrix id and MapMan annotation to bin name, pathway and description of gene model. Analysis of results are presented in individual spreadsheets for all MapMan bins and sorted for different leaf %RWC for the three lines. In addition, probes from all the bins which showed expression changes at the earliest stress point of 82% are highlighted for the three lines in spreadsheet ‘early changes’ and sorted for the three lines in subsequent spreadsheets. A summary of two probes showing the largest changes measured at the early stage of 82%RWC for each line is presented in spreadsheet ‘summary top changes early’.
(XLSX)

File S6 **MapMan analysis of ANOVA ‘** ***no effect*** **208 probes’** **group.** Probes, post initial filtering, showing no difference in expression between the three lines Lahn, Cham1 and RIL2219, or in response to the stress from data in File S1. Probes are presented by Affymetrix id and MapMan annotation to bin name, pathway and description of gene model.
(XLSX)

File S7 **MapMan annotation of PCo results.** Mapman analysis of probes responsible for maximum separation observed, as defined by F-tests for the correlation between probes and principal coordinates (PCos), following PCo applied to the RWC-interpolated data for the 19062 probes derived from 54 samples (three replicates of each of six RWC levels by three wheat lines). Individual spreadsheets show annotation for the probes relevant for the first three principal coordinates retained from the analysis (PCo1, 2, 3). Probes are presented by Affymetrix id and MapMan annotation to bin name, pathway and description of gene model.
(XLSX)

File S8 **The contribution of allelic differences in gene sequences to probe hybridisation for probes annotated to transcription factors.** ANOVA for each perfect match probe and the significance of the ratio changes across lines was calculated in proportion to the corresponding mean probe-set abundance. The name of the probe and P value are given for the corresponding probe set taken from *line only*, *line+stress* and *Line x stress* ANOVA groups datasets from File S3,4,5 respectively focusing on MapMan bin 27.
(XLSX)
