# Supplementary figures and images for: Systems Responses to Progressive Water Stress in Durum Wheat
Source: PLoS One. 2014 Sep 29;9(9):e108431. doi: 10.1371/journal.pone.0108431 (PMC4180936; doi:10.1371/journal.pone.0108431)

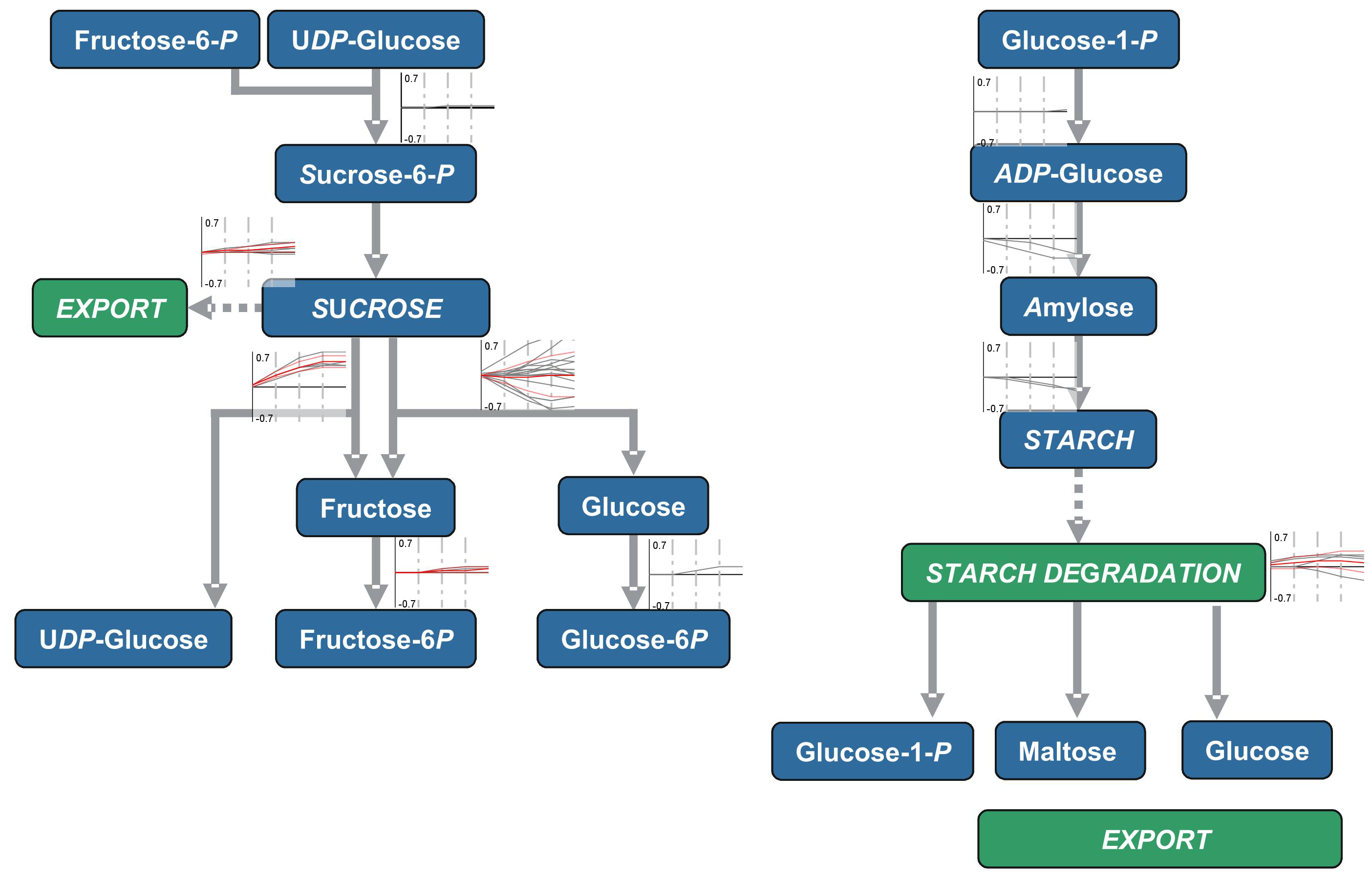

Supplement: Figure S1 — Expression of genes in sugar metabolism during stress. Expression of probes annotated to MapMan bin 2 as a function of decreasing leaf %RWC. ANOVA Dataset and visualisation as for Figure 3. (TIF) [file pone.0108431.s001.tif]
